# AI-Driven Advances in Parkinson’s Disease Neurosurgery: Enhancing Patient Selection, Trial Efficiency, and Therapeutic Outcomes

**DOI:** 10.3390/brainsci15050494

**Published:** 2025-05-09

**Authors:** José E. Valerio, Guillermo de Jesús Aguirre Vera, Maria P. Fernandez Gomez, Jorge Zumaeta, Andrés M. Alvarez-Pinzon

**Affiliations:** 1 Neurosurgery Innovation and Technology Division, Latinoamerica Valerio Foundation, Weston, FL 33331, USA; jevalerio@jvaleriomd.com (J.E.V.);; 2 Department of Neurological Surgery, Palmetto General Hospital, Miami, FL 33016, USA; 3 Neurosurgery Oncology Center of Excellence, Department of Neurosurgery, Miami Neuroscience Center at Larkin, South Miami, FL 33143, USA; 4 GW School of Business, The George Washington University, Washington, DC 20052, USA; 5 Tecnológico de Monterrey School of Medicine, Monterrey 64710, Mexico; 6 The Institute of Neuroscience of Castilla y León (INCYL), Cancer Neuroscience, University of Salamanca (USAL), 37007 Salamanca, Spain; 7 Cellular Theraphy Program, Universidad de Granada, Hospital Real de Granada, 18071 Granada, Spain; 8 Institute for Human Health and Disease Intervention (I-HEALTH), Florida Atlantic University, Jupiter, FL 33431, USA

**Keywords:** Parkinson’s disease, artificial intelligence, machine learning, clinical trials, neurodegenerative disorders

## Abstract

Parkinson’s disease (PD) is a progressive neurodegenerative disorder marked by motor and non-motor dysfunctions that severely compromise patients’ quality of life. While pharmacological treatments provide symptomatic relief in the early stages, advanced PD often requires neurosurgical interventions, such as deep brain stimulation (DBS) and focused ultrasound (FUS), for effective symptom management. A significant challenge in optimizing these therapeutic strategies is the early identification and recruitment of suitable candidates for clinical trials. This review explores the role of artificial intelligence (AI) in advancing neurosurgical and neuroscience interventions for PD, highlighting the ways in which AI-driven platforms are transforming clinical trial design and patient selection. Machine learning (ML) algorithms and big data analytics enable precise patient stratification, risk assessment, and outcome prediction, accelerating the development of novel therapeutic approaches. These innovations improve trial efficiency, broaden treatment options, and enhance patient outcomes. However, integrating AI into clinical trial frameworks presents challenges such as data standardization, regulatory hurdles, and the need for extensive validation. Addressing these obstacles will require collaboration among neurosurgeons, neuroscientists, AI specialists, and regulatory bodies to establish ethical and effective guidelines for AI-driven technologies in PD neurosurgical research. This paper emphasizes the transformative potential of AI and technological innovation in shaping the future of PD neurosurgery, ultimately enhancing therapeutic efficacy and patient care.

## 1. Introduction

Parkinson’s disease is one of the most common neurological conditions worldwide, and the fastest growing in terms of prevalence, deaths according to the Global Burden of Disease (GBD) 2015, and the leading cause of disability among neurological disorders [1,2]. It is a progressive and deteriorating disease that presents with both motor and non-motor symptoms. The global incidence of Parkinson’s disease was estimated over 8.5 million people in 2019 by the World Health Organization (WHO), with 5.8 million disability-adjusted life years (DALYs), increasing 81% since 2000, 329,000 deaths, increasing 100% since 2000 [3]. The global burden of Parkinson’s disease has increased from 2.5 to 6.1 million in the last three decades [4]. While dopaminergic therapies are effective in managing symptoms like bradykinesia, they are not as effective for symptoms like postural inability and gait difficulties, which commonly appear in patients 10–15 years after symptom onset [5]. This highlights the need for new treatment approaches that improve patients’ quality of life.

Surgery, particularly deep brain stimulation, has effectively treated medication-refractory tremors, especially in patients without motor fluctuations. Even after optimization with levodopa, patients with tremors can benefit from deep brain stimulation [6]. Common targets for this therapy are the subthalamic nucleus (STN) or the globus pallidus internus (GPi), and it significantly improves motor symptoms in the short and long term. A common consensus among professionals is that deep brain stimulation significantly enhances the quality of life for patients with movement disorders [7]. However, patients may experience stimulation-induced side effects such as dysarthria, imbalance, and dyskinesia, which may require adjustments in stimulation [8], particularly in the initial stage following surgery [9]. This proves the necessity of ever-evolving treatment refining, which uses rising technologies to improve patients’ health.

The current trend is cutting-edge technologies that use Artificial Intelligence, this technology can help to address critical challenges that healthcare providers face. AI’s ability to learn and analyze patterns from large data sets, identifying patterns that may otherwise go unnoticed [10]. AI is a powerful tool that can complement humans. When used under human supervision, AI has been shown to improve patient outcomes and reduce errors in clinical settings [11].

This review aims to describe the current advancements in diagnosing Parkinson’s disease, particularly in the early stages of the disease. Currently, there are no biomarkers for the early stages of Parkinson’s disease or for tracking its progression [12]. Artificial intelligence has the potential to significantly enhance symptom monitoring and potentially define molecular subtypes through global datasets [13]. This review also aims to critically evaluate how early-phase trials are conducted and to establish alternatives to refine the design and execution of neurosurgical studies in Parkinson’s disease.

## 2. Methods

A systematic literature review was conducted in alignment with PRISMA guidelines to identify peer-reviewed studies investigating the application of artificial intelligence (AI) and machine learning (ML) in Parkinson’s disease (PD). The primary data source was PubMed, supplemented by reference mining of key review articles. Boolean logic was used with the following search terms: (“Parkinson’s Disease” OR “PD”) AND (“Artificial Intelligence” OR “Machine Learning”). The search was restricted to English-language articles published between January 2012 and March 2025. The screening process was guided by well-defined inclusion criteria, focusing on studies assessed independently by two reviewers (J.E.V. and G.d.J.A.V.), with a third reviewer (A.M.A.-P.) consulted to resolve any conflicts.

Eligible studies included those that applied AI/ML methodologies to enhance diagnostic accuracy, enable early disease screening, inform patient stratification, or guide therapeutic interventions in PD. Specifically, studies were included if they employed supervised or unsupervised learning models utilizing clinical data, neuroimaging (MRI, PET), electrophysiological signals, or biomarker profiles. Articles were required to report statistical performance metrics such as accuracy, sensitivity, specificity, area under the ROC curve (AUC), or F1-score. Further inclusion required validation of models through established techniques, including k-fold cross-validation, bootstrapping, or external dataset testing. Studies were excluded if they did not incorporate a clear AI/ML methodology, lacked empirical results, or focused solely on device development without clinical application. Data extracted from eligible studies included algorithm types (e.g., random forests, neural networks, support vector machines), input feature sets, preprocessing pipelines, outcome variables, and model evaluation methods. To assess methodological rigor and potential bias, we applied a modified version of the PROBAST tool. Findings were stratified by application domain and summarized both qualitatively and quantitatively, with meta-analytical techniques employed when appropriate to synthesize model performance across comparable studies.

The screening process prioritized machine learning-enhanced patient sampling, emphasizing datasets from PD patients where AI technologies facilitated pattern recognition, neuroimaging analysis (e.g., MRI, PET, and functional imaging), and real-time neurophysiological monitoring. Particular emphasis was placed on studies demonstrating the role of AI in preoperative planning for deep brain stimulation (DBS), precision targeting for focused ultrasound (FUS), and optimization of closed-loop neuromodulation systems.

## 3. Pathomechanisms Leading to Parkinson’s Disease: An AI-Driven Perspective

The pathogenesis of PD is driven by a complex interplay of genetic, molecular, and environmental factors, leading to progressive neurodegeneration. Central to PD pathology is the degeneration of dopaminergic neurons in the substantia nigra and the accumulation of alpha-synuclein aggregates, both of which have been linked to immune dysfunction and neuroinflammatory pathways [13,14,15,16,17].

AI-powered systems are increasingly being deployed to model the molecular and cellular pathways underlying PD, offering advanced insights into:

Neuroinflammation Modeling: AI-driven simulations analyze the activation of microglia and astrocytes, which respond to alpha-synuclein aggregates and other neuronal signals by secreting pro-inflammatory cytokines and reactive oxygen species. These models help predict neurodegenerative progression by assessing inflammasome pathway activation (NLRP3) and caspase-1-mediated cytokine production.

Genetic and Environmental Risk Mapping: Deep learning frameworks integrate genomic, transcriptomic, and exposomic data to elucidate interactions between environmental neurotoxins (e.g., MPTP, rotenone) and immune-mediated neurodegeneration. These computational approaches refine risk stratification models, identifying high-risk patients before clinical manifestation.

Neuropeptide Dysregulation and Sleep-Wake Cycle Prediction: AI-based neuroimaging and cerebrospinal fluid (CSF) analysis have revealed the dysregulation of orexin neurons, which are implicated in sleep disturbances, autonomic dysfunction, and neurodegeneration. Predictive models assess orexin-A deficiency and its impact on oxidative stress and brain-derived neurotrophic factor (BDNF) expression, offering potential therapeutic targets [17,18,19,20].

## 4. AI and Anatomical and Structural Changes

Identifying the specific morphological structure implicated in the pathogenesis of Parkinson’s Disease in each patient is an important clinical feature. Due to variations in disease progression, the pace of cognitive deficit, neuropsychiatric symptomatology, and responses to different treatment modalities may vary. Clinical decision-support systems could be a helpful alternative to assist clinicians in decision-making for patients [21,22,23].

A study utilized a wide range of morphological features alongside clinical test scores to develop a machine-driven method for differentiating Parkinson’s Disease and progressive supranuclear palsy (Richardson’s syndrome), which is considered the most common phenotype of progressive supranuclear palsy [24]. Early stage and accurate differentiation diagnosis of both conditions is difficult for clinicians due to overlapping symptoms [25]. The classification method utilized by the study involved a feature selection method followed by a classification algorithm. After the ranking, a support vector machine (SVM) with a linear kernel was implemented to manage the balance, reducing misclassification and minimizing errors. The result was a combination of subcortical morphological properties, including regional brain volumes, brain surface area, and the ratio of brain surface area to volume, which can distinguish between Parkinson’s Disease and progressive supranuclear palsy [26].

The thalamus plays a crucial role in the pathogenic process and clinical disease onset of symptoms. A systematic review aimed to elucidate the morphological changes in the subthalamic nuclei in Parkinson’s disease patients. Using support vector machines, a type of machine learning, researchers developed an algorithm that could predict the UPDRS III score for limb bradykinesia, axial akinetic score, and UPDRS III improvement. Applying pattern analyzing techniques to MRI revealed morphological changes, including increased volumes of bilateral thalami enlargement and atrophy of the left thalamic subnuclei [27].

## 5. AI and Utilization of Plasma Proteomics on Predicting Parkinson’s Onset

The clinical heterogeneity of Parkinson’s presents a significant challenge for developing neuroprotective strategies that prevent disease progression. One of the main difficulties is the lack of measurable biomarker indicators [28]. This has led to calls for markers that can impartially assess Parkinson’s disease phenotypes and potential therapeutic pathways and identify biomarkers associated with the disease’s clinical pathophysiology [29]. Recent evidence suggests that cerebrospinal fluid (CSF) and blood biomarkers may have diagnostic and prognostic value by accurately reflecting the underlying mechanisms of Parkinson’s disease. For example, specific biomarkers such as α-synuclein, lysosomal enzymes, amyloid and tau pathology indicators, and neurofilament light chain show promise for early diagnosis of Parkinson’s disease [30].

A study used mass spectrometry-based proteomic phenotyping to identify a panel of blood biomarkers in Parkinson’s disease. The study characterized a group of De novo Parkinson’s disease patients and healthy controls through collection protocols, and a machine learning model was trained with this information. Utilizing a regression pattern, the model identified a panel of proteins that could distinguish between De novo Parkinson’s disease and control samples with 100% accuracy based on the expression of eight proteins (GRN, MASP2, HSPA5, PTGDS, ICAM1, C3, DKK3, and SERPING1). This biomarker selection revealed a distinctive signature of protective and detrimental mechanisms, shedding light on the pathways that trigger oxidative stress and neuroinflammatory responses, key factors leading to α-synuclein aggregation and Lewy body formation. This underscores the importance of machine learning in early identification and diagnosis of neurodegenerative diseases, as the model was able to identify this signature in the prodromal non-motor phase (stage 2 NSD), occurring up to 7 years before the onset of motor or cognitive symptoms (stage 3) [31].

The current diagnosis of Parkinson’s Disease heavily relies on clinical and motor symptoms. Cognitive impairment is particularly prevalent, and in the early stages of the disease, it could arise in an insidious form such as mild cognitive impairment in up to 25% of newly diagnosed patients [32,33]. Cognitive decline tends to worsen, leading to significant disability. Parkinson’s Disease patients with mild cognitive impairment are six times more likely to develop dementia than matched controls, with a prevalence of 80% after 15 to 20 years of living with the disease [34]. A dual syndrome hypothesis was established regarding two different structural and neurotransmission components involved in cognitive decline [35]. The first correlated to the reduction of dopamine levels in the basal ganglia, ultimately causing a disturbance in the cortico-basal ganglia-thalamus-cortical (CBGTC) loops [36,37].

Event-related potentials (ERPs), derived from scalp-recorded electroencephalography (EEG), represent time-locked neural responses to specific sensory, cognitive, or motor events. These signals are typically extracted by averaging EEG activity across repeated stimulus presentations to isolate phase-locked neural components associated with task-relevant brain activity. ERPs are believed to reflect the summation of postsynaptic potentials generated by large populations of synchronously active pyramidal neurons within the cerebral cortex. Recent clinical investigations have demonstrated the utility of ERP features as neurophysiological biomarkers for the diagnosis and monitoring of Parkinson’s disease (PD), particularly in patients undergoing deep brain stimulation (DBS). The integration of artificial intelligence (AI) and machine learning (ML) algorithms into ERP analysis has enabled advanced signal processing and feature extraction techniques that enhance diagnostic sensitivity and specificity. For instance, supervised learning models such as support vector machines (SVMs), random forests, and convolutional neural networks (CNNs) have been employed to classify ERP waveforms and temporal dynamics associated with PD-related neural dysfunction. Feature engineering approaches—including principal component analysis (PCA), wavelet transforms, and independent component analysis (ICA)—further aid in dimensionality reduction and noise minimization, optimizing the performance of classification algorithms. This AI-enhanced ERP methodology offers a noninvasive and scalable approach for the early detection and longitudinal monitoring of PD. As an adjunct to clinical assessments and neuroimaging, ERP-based ML models show promise in capturing subtle alterations in brain dynamics associated with disease progression or therapeutic modulation through DBS. One recent small-scale study evaluated the real-time application of AI in ERP signal interpretation, potentially enabling personalized neuromodulation strategies and precision diagnostics in movement disorders [38,39].

A recently developed study used high-resolution EEG recording to analyze brain activity during Go/No-Go (VGNG) and auditory Oddball (AOB) cognitive task performance in patients with early and advanced-stage Parkinson’s Disease. Event-related potentials were analyzed through Brain Network Analytics technology to produce portrayals of brain activity [40]. The study identified potential markers for early-stage Parkinson’s disease using a machine-learning algorithm. The analysis resulted in 15 distinct features that can distinguish early-onset Parkinson’s disease from healthy controls. Five of these features are related to three specific cognitive functions: early sensory processing (P50 amplitude, latency), information filtering (amplitude and topographic similarity), and response-locked activity. These findings are promising and could be an important tool for early diagnosis. Further research with larger patient samples and testing on premotor phase patients can help fully understand its potential as an auxiliary early diagnostic tool [41].

Cerebrospinal fluid (CSF) is a commonly tested biomarker collected from patients with neurological symptoms. Researchers have suggested that CSF closely reflects the changes in Parkinson’s Disease patients. For example, there are significant increases in ion densities like iron, while ferritin levels decrease over time. CSF has the potential to be an important biomarker for tracking neurodegeneration over time [42]. This becomes relevant when considering the genetic factors of Parkinson’s disease. While most cases of Parkinson’s are idiopathic, there is evidence of a genetic pattern linked to the illness [43].

Mutations in the leucine-rich repeat kinase 2 (LRRK2) gene are the most common cause of autosomal dominant Parkinson’s disease. Understanding the pathophysiological mechanisms could lead to therapeutic targets for genetically triggered Parkinson’s disease. Understanding the pathophysiological mechanisms could lead to therapeutic targets for genetically triggered Parkinson’s disease. Identifying this biomarker could facilitate its potential inhibition [44,45,46].

A research study conducted proteomics analysis of CSF from healthy controls, Parkinson’s disease (PD) patients with and without the LRRK2 G2019S mutation, and individuals with non-manifesting LRRK2 G2019S. This analysis used mass spectrometry (MS) to identify necessary biomarkers. This was done through a predictive machine learning model developed to assist in classifying Parkinson’s disease based on bioelement abundance. Machine learning-based proteomics is a powerful technology for identifying differences in protein abundance. However, CSF generally has low protein concentrations and an expanded dynamic range of disturbances [47,48,49]. By applying co-variate (ANCOVA) analysis and machine learning, the last one benefits from large subsets of samples. The tool used was OmicLearn, which assisted in executing the data analysis, model execution, and producing the plots and charts [50]. Researchers identified specific proteins correlated with clinical scores and enhanced neuroinflammation, once again demonstrating the critical role of machine learning in pattern elucidation [51].

The first multicenter trial has been conducted to test for significant elemental signatures in CSF compared to control patients. This trial focused on the density of bio elements rather than the concentrations of biomolecules. Chemical elements do not degrade and remain stable at low and moderate temperatures. A predictive machine learning model was trained to classify Parkinson’s Disease Patients and matched controls based on predictions of the density of bio elements in CSF. The model used a radial kernel Support Vector Machine (SVM) algorithm, which was trained on preprocessed CSF levels of As, Fe, Mg, Ni, Se, and Sr from a recently discovered cohort. The study showed that the model had an area under the receiver operating characteristic curve of 0.76 for the differentiation of Parkinson’s Disease and Age-matched controls based purely on calculations made identifying the bio elements. Supporting the hypothesis of bio elements having the potential to distinguish among disease cohorts. However, further advancements are needed to understand factors contributing to a center bias, as presented in the study. Nonetheless, this study serves as a precedent for the potential of machine learning in discovering patterns of information [52,53,54,55].

## 6. AI and Other Mutations Related to PD Pathogenesis

The pathogenesis of Parkinson’s disease (PD) is influenced by a complex interplay between genetic predisposition and environmental factors. Recent advancements in artificial intelligence (AI) and computational genomics have facilitated the identification of novel gene variants implicated in PD, significantly enhancing our understanding of disease mechanisms. Machine learning algorithms and big-data approaches have enabled large-scale genome-wide association studies (GWAS) and deep-learning-driven mutation analyses, uncovering both established and newly emerging genetic risk factors.

Recent studies have identified several putative or confirmed PD-associated genes, including ANK2, DNAH1, STAB1, NOTCH2NLC, UQCRC1, ATP10B, TFG, CHMP1A, GIPC1, KIF21B, KIF24, SLC25A39, SPTBN1, and TOMM22. However, due to the novelty of these findings, the specific molecular mechanisms linking these mutations to PD remain inconclusive, necessitating further investigation through AI-assisted proteomic and transcriptomic modeling. One notable genetic variant, TMEM175, has been linked to dysfunctional lysosomal K+ channels and has shown a significant association with PD in Italian population studies. AI-driven functional studies suggest that TMEM175 variants impact autophagic-lysosomal proteolytic flux, leading to impaired protein degradation pathways and increased unfolded protein response activation—both crucial contributors to PD pathogenesis.

Mutations in the *RAB32* gene have recently been implicated in familial Parkinson’s disease (PD), particularly through modulation of LRRK2 kinase hyperactivity—a known molecular driver of neurodegeneration. The c.213C  >  G/p.S71R variant has emerged as a high-risk allele, demonstrating an odds ratio of 65.5 in familial PD cohorts. Recent studies using AlphaFold-based protein structure prediction and AI-guided protein-ligand interaction mapping have validated its pathological significance, revealing altered conformational states that promote aberrant kinase signaling cascades in dopaminergic neurons. To further characterize these mutations, machine learning classifiers such as XGBoost, support vector machines (SVMs), and random forest algorithms have been deployed on large genomic datasets to distinguish pathogenic variants from benign polymorphisms. These models integrate multi-omic data—including transcriptomic, proteomic, and epigenetic features—enhancing predictive accuracy. In parallel, unsupervised learning approaches like hierarchical clustering and autoencoders have been applied to stratify patients by mutational burden and clinical phenotype [6,55,56,57,58,59,60].

This AI-enhanced framework for variant interpretation not only refines risk classification but also identifies novel therapeutic targets through graph-based deep learning models and network propagation algorithms. These advancements are accelerating precision medicine in PD, enabling earlier identification of at-risk individuals and the development of genotype-guided interventions, including tailored neurosurgical strategies and kinase-targeted therapies [56].

## 7. Disease Progression Patterns

Parkinson’s disease can be classified primarily based on the age of onset of the disease. Models have described distinct needs and characteristics of patients with young onset age [57]. Changes in speaking patterns and handwriting strokes are potential parameters to consider when attempting to diagnose this disorder early [58]. Efforts to classify Parkinson’s disease subtypes have focused on tremor-dominant subtypes, as well as characteristics of postural instability and gait-dominant subtypes [59].

Research in this field has led to new ways of predicting the development of Parkinson’s disease. Baseline clinical characteristics, cerebrospinal fluid samples, and imaging techniques have been used to predict changes in MDS-UPDRS and dopamine-transporter binding. While not highly effective on their own, accuracy increased when short-term changes were added to the prediction model [60]. This has raised the question of whether weather patterns could have a significant correlation with Parkinson’s disease progression. If this were the case, they could be standardized to aid machine learning models in predicting patterns through large data clusters [61,62,63,64,65].

## 8. Current Treatment

The management of Parkinson’s disease (PD) is increasingly benefiting from artificial intelligence (AI) and machine learning (ML) applications that personalize therapeutic strategies. Traditional pharmacologic regimens anchored by levodopa remain essential for controlling core motor symptoms. However, ML models, including supervised learning algorithms such as random forests and support vector machines, are now being deployed to predict individual response trajectories, identify early signs of levodopa-induced dyskinesia, and inform adjustments to adjunct therapies like MAO-B inhibitors or dopamine agonists [66,67,68,69,70].

Advanced ML frameworks, including convolutional neural networks (CNNs) and ensemble learning models, are applied to high-dimensional neuroimaging (e.g., MRI, PET) and multi-omic biomarker datasets to stratify patients based on disease phenotype and projected progression. This stratification enables more precise identification of candidates for neurosurgical interventions like deep brain stimulation (DBS) or focused ultrasound (FUS). These AI-driven insights support early, evidence-based clinical decision-making that aligns with the patient’s unique neurobiological profile. In parallel, wearable biosensors integrated with deep learning algorithms and time-series analysis are enabling real-time monitoring of motor fluctuations and non-motor symptomatology. These tools allow for dynamic, closed-loop treatment systems where stimulation parameters or pharmacologic regimens are adapted in response to continuous physiological feedback. Such AI-guided precision neurology enhances treatment efficacy, reduces side effects, and fosters proactive care in both clinical and remote settings.

## 9. AI and Clinical Trials

Patient screening for clinical trials is a critical component in the successful development of therapeutic and neurosurgical interventions for Parkinson’s disease (PD). Accurate and efficient patient selection ensures that clinical trials can identify the right candidates who are most likely to benefit from the intervention while minimizing risks. This process is particularly crucial in neurosurgical trials, where precision in targeting specific patient subgroups—such as those eligible for deep brain stimulation (DBS) or focused ultrasound (FUS)—can significantly influence trial outcomes. Traditionally, patient screening relies heavily on clinical criteria, including symptom severity, disease stage, and the presence of comorbidities. However, these conventional methods can be labor-intensive and subjective, often leading to delays in recruitment and inconsistent trial results. The advent of artificial intelligence (AI) and machine learning (ML) technologies has revolutionized the patient screening process, introducing more sophisticated and data-driven approaches to clinical trial recruitment [19,21,55].

AI-driven systems utilize large datasets, including patient demographics, clinical histories, neuroimaging, genetic profiles, and biomarkers, to create comprehensive models of PD progression and treatment response. By analyzing patterns and trends within these datasets, AI algorithms can identify patients who meet specific criteria for inclusion in therapeutic and neurosurgical trials, such as DBS or FUS candidates. These systems can also predict the likelihood of a patient’s response to treatment, enabling a more personalized approach to trial participation [21,34,71].

In therapeutic trials, AI tools can support screening by analyzing both motor and non-motor symptom profiles and selecting patients based on a broader spectrum of disease features. For instance, ML algorithms can assess cognitive decline, mood disturbances, and autonomic dysfunction, which are essential considerations for the selection of appropriate patients for pharmacological or combined therapeutic interventions. Similarly, AI-driven predictive models can offer insight into disease trajectories, allowing researchers to better identify individuals who would benefit most from the trial’s investigational therapies [19,71,72,73,74,75].

In neurosurgical trials, patient screening benefits from AI’s ability to analyze complex neuroimaging data, such as functional magnetic resonance imaging (fMRI) or positron emission tomography (PET) scans. These advanced imaging modalities, when coupled with AI, allow for the precise identification of brain regions affected by PD, aiding in the selection of patients who are likely to respond to neurosurgical interventions like DBS or FUS. Moreover, AI systems can integrate real-time monitoring data, providing continuous assessments of patient eligibility and ensuring that the right candidates are selected throughout the course of the trial. Additionally, AI enhances patient stratification by incorporating genetic and biomarker data, further refining patient selection and ensuring that participants are well-suited for the intervention under investigation. Biomarkers, including those identified through liquid biopsy, can be particularly useful in screening for underlying pathophysiological mechanisms that may influence treatment efficacy. This is especially important in trials exploring novel neurosurgical technologies that target specific PD-related brain networks or pathways [75,76,77,78,79,80,81].

Despite these advances, integrating AI into patient screening in clinical trials poses challenges, including the need for robust validation of algorithms and the establishment of ethical frameworks for data usage. There is also a critical need for data standardization to ensure that AI-driven systems can function effectively across diverse populations and clinical settings. To overcome these challenges, collaboration between clinicians, AI experts, and regulatory bodies is necessary to establish best practices and guidelines for the ethical implementation of AI technologies in PD clinical trials [82,83,84,85,86,87,88,89,90].

AI and machine learning are reshaping patient screening in clinical trials for both therapeutic and neurosurgical interventions in Parkinson’s disease. By leveraging large-scale data and predictive models, these technologies can significantly improve the precision and efficiency of patient recruitment, ultimately enhancing the likelihood of success in clinical trials and optimizing therapeutic outcomes for PD patients.

## 10. Neurosurgical Interventions and Their Significance in the Context of PD Subtypes

The management of medication-resistant tremors in Parkinson’s Disease (PD) has evolved significantly, offering several surgical and ablative approaches tailored to individual patient needs. Patients who experience intolerable side effects from pharmacological treatments or continue to have tremors despite optimized medication regimens are prime candidates for surgical intervention [61,91,92,93,94]. Focused ultrasound thalamotomy has emerged as a viable alternative to deep brain stimulation (DBS) and traditional lesional surgery, thus expanding the eligibility for such interventions, particularly among elderly patients and those with significant comorbidities. Bilateral subthalamic nucleus (STN) stimulation has demonstrated robust efficacy in alleviating motor symptoms and enhancing quality of life [62,93], showing an impressive 82% reduction in resting tremor during “off” medication periods, as evidenced by both randomized controlled studies and long-term follow-ups. Additionally, STN stimulation has proven effective in patients with fluctuating conditions and dyskinesias, sustaining its therapeutic benefit for over five years [61,64,94,95,96,97,98,99].

DBS is specifically indicated to certain subtypes of PD, especially in patients with motor fluctuations refractory to pharmacological therapy. DBS has shown a high effectivity in Tremor-Dominant Type (TDT) assessment, with studies showing that patients with DBS experience significant improvement in tremor control, despite there being studies which suggest that GPi DBS offers better gait results for this specific subtype [66,91,92,93,94,95,96,97,98,99]. Akinetic-Rigid Type (ART) has also been shown to benefit from DBS, with evidence showing this when subthalamic nucleus (STN) is targeted. This is true when compared to GPi DBS, providing significant improvements in rigidity, akinesia, and posture and gait disorders [68]. Mixed type PD treated through DBS has been established to generally lead to a significant motor symptoms improvement, specifically in tremor, rigidity, and bradykinesia, this is particularly true when there is an “off” medication state [99,100,101,102].

The globus pallidus internus (GPi) has also been described as an alternative target for those with medication-refractory motor complications, exhibiting comparable outcomes to STN stimulation over one and ten-year follow-ups, though with generally lesser motor effects and insufficient reduction of PD medications [61,70,101]. Thalamic ventral intermediate nucleus (VIM) stimulation has been shown to provide sustained relief from unilateral limb tremors, demonstrating a greater than 60% improvement over ten years. While VIM stimulation cannot address other motor symptoms of PD, it remains a suitable option, especially for elderly patients due to its favorable safety profile [61,102,103,104].

Historically, lesional surgery, including radiofrequency thalamotomy, provided an initial strategy for treating refractory tremors from the 1960s to the 1980s. However, advancements have favored the use of DBS, particularly with VIM stimulation, which offers superior functional outcomes and a lower rate of adverse events. Gamma knife thalamotomy and magnetic resonance-guided focused ultrasound (FUS) thalamotomy present newer modalities, the latter distinguished by its non-invasive nature and real-time monitoring capabilities [74,105].

## 11. DBS in Comparison with FUS and GK Therapy for Parkinson’s Disease

Deep Brain Stimulation (DBS), MRI-guided Focused Ultrasound (MRgFUS), and Gamma Knife Radiosurgery (GKRS) represent advanced neurosurgical modalities for managing Parkinson’s disease (PD), each with distinct mechanistic frameworks and clinical indications. DBS remains the most adaptable, offering programmable stimulation via implanted electrodes in targets such as the subthalamic nucleus (STN). Its reversibility and long-term efficacy make it ideal for younger, cognitively intact patients. MRgFUS, by contrast, provides non-invasive, incisionless lesioning of thalamic or subthalamic regions, with rapid tremor relief in tremor-dominant PD cases. GKRS delivers focused radiation for selective ablation, particularly for patients contraindicated for DBS or FUS, albeit with delayed symptomatic improvement. Artificial intelligence is increasingly critical in tailoring neurosurgical interventions to individual patients. AI-integrated electronic medical record (EMR) systems, leveraging supervised learning algorithms, synthesize clinical, radiologic, and genotypic data to guide modality selection—balancing efficacy, risk, and patient-specific neurological profiles. Predictive modeling tools such as gradient boosting and neural networks assess likelihoods of adverse events or treatment failure, enabling preoperative stratification and informed decision-making. These ML-enhanced pipelines enhance precision in recommending DBS for drug-resistant motor symptoms, FUS for unilateral tremor, or GKRS for medically frail individuals.

Furthermore, AI-driven platforms facilitate real-time analytics from wearable sensors and longitudinal follow-ups to monitor post-intervention outcomes. By incorporating reinforcement learning and anomaly detection, these systems dynamically flag deteriorating motor function or emerging complications, informing parameter adjustments or re-intervention timing. AI-guided trial matching algorithms also support enrollment in ongoing DBS or FUS clinical studies, expediting access to novel therapeutics. Altogether, the synergy of advanced neurosurgical technologies and AI-based personalization is redefining the clinical pathway for PD patients [6,87].

## 12. The Role of AI in Improving Deep Brain Stimulation

Recent literature has shown that deep learning and other artificial intelligence (AI) technologies have increasingly played a key role as a predictor for optimal deep brain stimulation parameters for Parkinson Disease [81,82,83,84,85,86,87]. This section will review current clinical trials incorporating AI and assess the most important findings from each, detailing insights on the improvements of diagnostics and treatment through Artificial Intelligence Technologies.

One significant area of advancement is clinical decision-making supported by AI. Recent developments have led to the initial FDA approvals for AI-based deep brain stimulation (DBS) systems, allowing decision-aid models to be approved to play a clinical role. The techniques associated with AI have modernized significantly, evolving into three main categories: analysis and segmentation, image reconstruction, and, more recently, methods for artifact correction and noise reduction. These advancements stem from progress in deep learning algorithms, enhancements in localized processing capabilities, and an increasing recognition of deep learning’s potential in medical imaging [88,91,105].

Deep brain stimulation (DBS) is an effective treatment for Parkinson’s disease (PD), and its clinical outcomes have been further refined by the evolving role of artificial intelligence (AI). Recent applications of AI in this context focus on advancing neuroimaging technologies and predicting optimal stimulation parameters [92,93,94,95,96,97,98].

One study used AI with functional magnetic resonance imaging (fMRI) to predict the best DBS parameters for patients with PD. Researchers trained a machine learning model to determine optimal stimulation settings based on fMRI patterns, achieving a precision of 88%. This approach could potentially reduce the need for outpatient visits to adjust DBS and provide an objective biomarker for treatment response [96,106].

Another study found that AI algorithms applied to reconstructed, postprocessed MRI images significantly improved image quality and diagnostic usefulness when assessing neuropsychiatric functions in PD patients undergoing DBS. With MRI images, the incorporation of AI-driven noise reduction strategies allowed better visualization and monitoring of brain changes related to DBS treatment. could effectively observe clinical changes in both motor and cognitive functions, potentially aiding clinical decision-making [99].

## 13. Algorithms for Reviewing MRI

Deep brain stimulation offers a significant opportunity for clinicians to improve a patient’s quality of life. However, it also presents many challenges, particularly in determining each patient’s proper settings and parameters. Setting up deep brain stimulation often requires multiple clinical visits to review numerous potential parameters to find the optimal setting for symptom relief and to minimize side effects [98,101,102,103,104,105]. This process puts significant financial strain on patients and healthcare systems and prolongs the time before patients can benefit from this treatment option [99,106]. Neuroimaging techniques have played a crucial role in enhancing surgical outcomes, especially in movement disorders like Parkinson’s disease. Diffusion tensor imaging allows for the anatomical segmentation of cortical areas and functional subregions within the deep target area [102].

Studies have used AI-based magnetic resonance imaging volumetry to compare possible structural differences between essential tremor and tremor-dominant Parkinson’s disease patients. This approach has helped to identify important anatomical and structural changes between the subtypes, showing variations in the occipital lobes, hippocampus, putamen, pallidum, and mesencephalon. Essential tremor patients showed decreased caudate nucleus and thalamic volumes. Despite the limitations of the study, this approach highlights the importance of Artificial Intelligence as a valuable tool for understanding clinical phenotypes and their underlying pathology, aiding in describing the benefits of targeting different areas when using therapeutic measures such as neurosurgical interventions [103].

Prospective fMRI data has been used with optimal standards to identify brain activity patterns with clinical benefits in Parkinson’s disease patients as an indicator of deep brain stimulation efficacy. The prediction of optimal deep brain stimulation settings was achieved through machine learning, which analyzed brain responsiveness patterns [104]. Another study explored the potential of achieving personalized deep brain stimulation through whole brain radiomics using machine learning techniques like regularized binary logistic regression (LR), Gaussian naïve Bayes (NB), K-nearest neighbors (KNN), highlighting the effectiveness of Artificial Intelligence as a tool for clinical decision support and providing personalized recommendations for Parkinson’s disease patients [104,105].

Tools for radiologic assessment have been developed. Imaging patterns of regional glucose metabolism have allowed for reliable differentiation between Parkinson’s disease (PD) and atypical Parkinsonian syndromes [106]. A meta-analysis of 24 studies analyzed the use of this tool, with 12 of the trials relying on Artificial Intelligence for automated identification. The analysis concluded that AI-mediated diagnostics had an accuracy comparable to that of a specialist radiologist, further supporting its role as an important adjunct tool for future diagnostics [104,105,106,107,108,109,110,111].

A summary of the main current evidence, detailing the description of the articles, key insights, and recommendations found in the reviewed manuscripts, can be found in Table 1.

## 14. Ethical and Regulatory Considerations in AI-Driven Trials

The integration of artificial intelligence (AI) into clinical trials, particularly in neurosurgery and Parkinson’s disease (PD) research, introduces a host of ethical and regulatory challenges. While AI promises to enhance patient selection, treatment personalization, and outcome prediction, these benefits come with significant concerns related to data privacy, informed consent, and algorithmic bias.

### 14.1. Data Privacy and Security

AI systems rely on large datasets containing sensitive patient information, which raises serious concerns about data privacy. Ensuring patient confidentiality is paramount, particularly when integrating data from diverse sources, such as electronic health records (EHRs), genomic data, and neuroimaging. Stringent measures must be implemented to prevent data breaches and protect patient anonymity. Additionally, data sharing between institutions must be secure and regulated to comply with laws like the Health Insurance Portability and Accountability Act (HIPAA) in the U.S. or the General Data Protection Regulation (GDPR) in the EU.

### 14.2. Informed Consent

AI’s involvement in clinical trials introduces complexity in the informed consent process. Patients must understand how their data will be used by AI systems, what role AI-driven algorithms play in decision-making, and the potential risks and benefits associated with AI-enhanced treatments. Researchers must ensure that AI’s decision-making role is clearly explained to patients, emphasizing that these systems are tools rather than final authorities in care. The issue of consent for secondary data use, such as for algorithm training, must also be addressed transparently.

### 14.3. Algorithmic Fairness and Bias

AI systems are susceptible to algorithmic bias if they are trained on data that lacks diversity or reflects historical disparities in medical care. These biases may result in inequitable treatment outcomes, particularly for minority populations. Ethical frameworks must evolve to ensure that AI models are fair, unbiased, and inclusive, reflecting the full spectrum of patient demographics. As AI-driven trials become more prevalent, ensuring equitable access to these technologies and minimizing the potential for discrimination is critical [88,89,90,91,92,93,94,95,96].

Morley et al. (2020) [112] emphasize that the ethical considerations in AI must move beyond the technology itself and encompass broader concerns, such as the social implications of its application in healthcare. This includes addressing how AI may inadvertently reinforce existing disparities and ensuring that vulnerable populations are not underserved by technological advancements.

### 14.4. Transparency and Accountability

As AI systems become integral to clinical decision-making, ensuring transparency in how algorithms arrive at conclusions is crucial for clinical accountability. Researchers must establish clear protocols for how AI-driven models are developed, validated, and tested before being used in patient care or clinical trials. It is essential that healthcare professionals understand the limitations of AI models and remain actively involved in the decision-making process.

### 14.5. Regulatory Oversight

Given the growing role of AI in clinical trials and neurosurgical interventions, regulatory bodies like the FDA, EMA, and other national health authorities must adapt and create specific guidelines for AI-based trials. This includes ensuring that AI technologies undergo rigorous testing to demonstrate safety, efficacy, and clinical relevance. Additionally, regulatory standards should be updated to encompass emerging AI technologies and address the ethical challenges they present.

## 15. Challenges and Future Research Directions

As AI technologies advance, future research must focus on validating these models in large, diverse patient populations across multiple clinical settings to ensure their robustness and clinical applicability. Rigorous testing will be required to confirm that AI-driven systems meet the highest standards of safety, efficacy, and generalizability. Additionally, refining these models to account for variabilities in disease progression and treatment responses across different subgroups is crucial for improving predictive accuracy. Furthermore, fostering multidisciplinary collaborations between AI specialists, neurosurgeons, clinicians, and researchers will be key to optimizing the integration of AI into clinical workflows. Such partnerships will address significant challenges in data privacy, algorithm transparency, and ethical concerns, ensuring that AI technologies are deployed responsibly and with patient welfare as a primary concern. Finally, securing regulatory approvals and ensuring clinical adoption will be vital steps toward realizing AI’s transformative potential in Parkinson’s Disease treatment and neurosurgical interventions.

## 16. Examples of Artificial Intelligence Architecture

It is important to list out and showcase the specific AI models which have been developed in current times. An illustrative case is the generation of an AI model that evaluates night-time breathing patterns in order to identify the PD and follow up its progression. The use of this model provided a high areas under the curve (AUC) of 0.90, additionally to performing with a 0.85 on external test sets, this is a testament of how non-invasive methods can diagnose PD effectively [12]. 

The system was implemented through a dedicated neural network designed with different modules: a devicedependent breathing feature extractor, a PD-specific representation encoding module, a binary classifier determining the disease presence, and a regression component to estimate the clinical severity according to the MDS-UPDRS scale. The ordered sequence of convolutional and recurrent operators of the feature extractor, computed time-series data to effectively encode respiratory dynamics over the night. These were then further processed through self-attention mechanism, which had the purpose to prioritize the most significant signal segments which were characterized of PD [12,13,14,15].

In order to improve the learning efficiency and avoid overfitting, the model was further trained under a multitask regimen with an additional objective: to also predict power distribution across EEG frequency bands from the same respiratory input pattern. This ancillary target enhanced model generalization and computational capture of neurological-related patterns. Additionally, the model utilized transfer learning to reconcile data between different sensor modalities. Such as wearable belts and contactless radio frequency monitors-and adversarial techniques to mitigate domain-specific bias. Regularization priors were used to ensure consistency across night recordings that was important in monitoring progression. The model’s test–retest reliability was high (ICC > 0.95) even with as few as 12 nights of data input, and it was found to be more sensitive to changes associated with disease than standard in-clinic measurements [12,99].

Specifically mentioning an example in the area of neuroimaging, a study utilized radiomic features extracted from 18F-FDG PET images combined with a support vector machine (SVM) classifier to distinguish PD patients from healthy controls. The model achieved accuracies of 90.97% and 88.08% on different test sets, highlighting the potential of machine learning techniques in enhancing diagnostic precision. The precise mechanism of implementation was through machine learning pipeline using radiomic parameters from 18F-fluorodeoxyglucose positron emission tomography (PET) images distinguishing Parkinson’s disease (PD) patients from controls was applied. The authors used a multistep analysis pipeline including spatial normalization of PET data into a standard brain template and subsequent Gaussian smoothing designed to improve signal-to-noise ratio [110,111,112,113,114].

Twenty-six ROIs were selected according to the anatomically and pathophysiologically significance and AAL atlas and previous studies. From these ROIs, >6000 radiomic features in high-dimension space including first-order intensity statistics, texture features based on matrices (i.e., gray-level co-occurrence matrix, or GLCM) and gray-level run length matrix (GLRLM), and wavelet transformed descriptors were extracted [114].

To deal with such a large feature space, a mass autocorrelation filter was firstly proposed to eliminate redundant ones, and Fisher score ranking was employed to select the top 30 most discriminatory features. Then the features were fed to Support Vector Machine (SVM) classifier with radial basis function kernels to draw a decision boundary between PD and controls. The performance of the classifier was outstanding, both in an external validation cohort with a mean accuracy rate of (JO contains this later for both with calculating all below for internal with GG) the internal test set (with a mean accuracy rate of). Especially, the low grey-level zone emphasis and long run high grey-level emphasis were robustly identified over repetitions and showed significant correlation with clinical scale (UPDRS and Hoehn & Yahr stage) implying both diagnostic and potential prognostic role [114].

EEG has also been exploited with deep learning methodologies. A multi-scale convolutional neural network (MCNN) model was proposed for analysis of EEG signals and the extraction of power spectral density (PSD) and phase-locked value (PLV) features. This model attained more than 99% of accuracy in the classification of PD patients, demonstrating its stability in extracting relevant neural patterns for the targeted disease [115].

The MCNN structure was formulated to combine spatial and frequency features in EEG. It was composed of two parallel convolutional subnetworks to independently handle 1-D (temporal) and 2-D (spatiotemporal) features derived from the EEG inputs. One of the subnetworks had a residual learning module, which was designed to alleviate degradation problem by increasing the network depth and promoting the feature propagation and gradient flow. All subnetworks consisted of convolution and max pooling layers with full connected layer at the end and drop out for regularization. The model was trained on mixed input matrices from PSD and PLV features across several frequency bands (δ to γ) computed through fast Fourier and Hilbert transform, respectively. It is worth to mention that training was performed on SGD with learning rate of 0.001. Subnetwork was trained by stochastic gradient descent (SGD) on a ratio (0.001). The performance was extensively validated using tenfold cross-validation on two independent EEG datasets (UC San Diego and Iowa), and the classification accuracy, sensitivity, specificity were all over 99% (in the γ-band) which were significantly better than the traditional machine learning baseline including SVM classifier [115,116,117].

In addition, AI method such as TFR and AlexNet convolutional neural network (CNN) has been used as the enemy-tactic to modalities for diagnosing EEG. This strategy has proved an effective tool in precisely recognizing complex EEG patterns associated with PD and allowing for early and reliable diagnosis. This study showcased a new architecture which combined Wavelet Scattering Transform (WST) with AlexNet CNN architecture to process the EEG signals [116].

The WST produces the high-resolution TFR images, which can well represent the temporal-spectral characteristics of nonstationary EEG recordings. These TFRs comprising oscillatory neural activity in various frequency bands are subsequently passed to a modified AlexNet CNN designed to identify spatial patterns at different scales. There are five convolutional layers followed by three fully connected layers in the CNN with ReLU activations, local response normalization, and dropout layers, which are used to improve the generalizable capability and the overfitting prevention, respectively. The final steps of the classification is made through a soft max layer classification. A novel aspect of this work was the detailed examination on a channel level that demonstrated that electrodes in the anterior frontal region (particularly AF4, AFz) were always associated with the highest classification accuracies. With this approach, the model outperformed the state-of-the-art results –99.84% accuracy on San Diego set and 95.79% on the Iowa set–and is able to be used as a tool for enlarger deploy mechanism [116,117].

In order to summarize the most important aspects of these models and their applications, look at Table 2 for a comprehensive overview of the AI architectures, their input modalities, diagnostic outputs, and performance metrics.

## 17. Conclusions

In conclusion, AI and machine learning are revolutionizing the management of Parkinson’s Disease and neurosurgery, enabling earlier diagnosis, personalized treatment plans, and enhanced surgical precision. By integrating advanced predictive models, real-time monitoring, and adaptive trial designs, AI holds the potential to transform patient care and accelerate therapeutic development. As these technologies continue to evolve, their application will lead to more effective, tailored interventions, improving outcomes and quality of life for individuals with Parkinson’s Disease. However, ongoing validation, ethical considerations, and integration into clinical workflows remain key challenges to fully realizing the potential of AI in this field.

## Figures and Tables

**Table 1:** Summary of current evidence.

Article	Article Description	Conclusion	Recommendations
Johnson, KA et al. [74]	This article provides an overview of the key findings from the Deep Brain Stimulation Think Tank XI. The focus is on the latest technologies in neuromodulation and new hypotheses regarding the integrative networks that support DBS treatment. The discussions also covered cutting-edge advances in other areas including physiology, translational neuromodulation, neuroethical dilemmas, algorithmic modeling, and artificial intelligence.	The meeting highlighted significant advancements in neuromodulation, particularly in understanding the mechanisms of Deep Brain Stimulation through animal models and human studies. It emphasized the importance of utilizing AI and large data-driven approaches to advance DBS as a widely used therapy.	The article recommends a continued emphasis on translational neuromodulation to gain a deeper understanding of this approach. It also suggests leveraging neurophysiological markers and machine learning algorithms to develop individualized treatments tailored to each patient, considering factors such as physiological changes, circadian rhythms, and sleep.
Purrer, V et al. [80]	The article discusses the issue of misdiagnosing patients with Parkinson’s disease and Essential tremor due to overlapping tremor features. The study examines if different tremor types have distinct brain characteristics. The researchers reviewed MRI scans of 61 patients with essential tremor and 29 with tremor-dominant Parkinson’s disease. They used Artificial Intelligence brain volumetry to compare various cortical and subcortical regions.	The study results indicate that essential tremor and tremor-dominant Parkinson’s disease share structural changes and show neurodegenerative mechanisms, particularly in the basal ganglia-thalamocortical. The study also found possible specific involvement of the thalamus in essential tremors.	The study suggests that AI-powered brain volumetry is a quick, reliable, and independent method to analyze brain volume. It helps understand specific patterns of brain atrophy in both discussed pathologies. The study underscores the need for further research to comprehend disease progression and to develop new treatment strategies.
Haliasos N et al. [82]	was to develop a machine learning-based predictive model for selecting patients for deep brain stimulation (DBS) using whole-brain white matter quantitative data from medical imaging and clinical variables. The study utilized machine learning methods such as logistic regression, support vector machine, naive Bayes, k-nearest neighbors, and random forest.	The study concluded that machine learning models can effectively predict the extent and progression of deterioration tailored to individual patients. It demonstrated high accuracy, particularly with the state-of-the-art Random Forest model, achieving up to 95% accuracy.	The article suggests further research into the potential of machine learning algorithms as auxiliary tools for clinicians in diagnostics and, importantly, for accurately predicting the progression of each patient’s illness and potential treatment responses, thus enabling personalized medicine.
Zhao, T et al. [81]	The study aimed to assess the effectiveness of 18F-FDG PET imaging in distinguishing between Parkinson’s Disease (PD) and Atypical Parkinsonian Syndromes (APSs).	The study found that 18F-FDG PET is highly accurate in differentiating PD from APSs. It also highlighted the significance of AI techniques, particularly deep learning, as powerful tools that can provide diagnostic performance comparable to traditional radiologist assessments.	The article acknowledges the potential impact of this differentiation in diagnosing PD from APDs. Additionally, it noted good accuracy for multiple system atrophy and progressive supranuclear palsy, suggesting potential for treatment response and disease monitoring.
Chahine, LM et al. [49]	The objective of this study was to investigate the key indicators that predict changes in motor and total MDS-UPDRS and DAT imaging within the first five years after being diagnosed with PD. This large-scale multicenter prospective cohort study was conducted internationally.	The results of the article demonstrate that initial and temporary changes in evaluations of motor disability (MDS-UPRRS) are the strongest predictors of long-term changes in the metrics used in the article. CSF and imaging measures in the early stages of PD indicated changes in MDS-UPDRS and dopamine transporter binding.	The main finding of this study is the potential for applying machine learning to Parkinson’s progression markers. This supports future efforts to establish reproducible and replicable models that utilize machine learning techniques applicable in clinical settings.
Talai, AS et al. [19]	The study aimed to address the challenge that clinicians encounter in distinguishing between Parkinson’s disease (PD) and progressive supranuclear palsy (PSP) due to their similar symptoms. The researchers evaluated the benefit of including additional morphological characteristics, in addition to clinical features, for the automated classification of PD and PSP-RS patients.	The study concluded that incorporating morphological features, along with clinical features, could be valuable for future computer-aided diagnostic protocols to differentiate between PD and PSP-RS patients.	The study also found that Support Vector Machines, a type of machine learning model, effectively achieved its purpose. It suggests that exploring other machine learning models such as random forests or neural networks could provide even better results when performing the classification process.
Lin, J et al. [64]	The article discusses the impact of magnetic resonance-guided focused ultrasound (MRgFUS) thalamotomy on the exploration of brain structure. It investigates the long-term changes in brain networks and identifies genetic changes related.	The study concludes that MRgFUS thalamotomy effectively reduces tremors in PD patients. However, it induces dynamic changes in the network topology of the brain. Making correlations with gene signatures	The authors recommend future studies to focus on the correlation between the structural network changes induced by MRgFUS thalamotomy and dopaminergic pathways. They also emphasize the importance of genetic mechanisms in the alteration of dopaminergic pathways.
Yasaka, K et al. [72]	The study aimed to determine if Parkinson’s disease can be distinguished from healthy controls by identifying neural circuit disorders using deep learning techniques and parameters.	The study found that PD can be differentiated from healthy controls by using a deep learning technique to analyze parameter-weighted connectome matrices.	It is recommended that further research be conducted on the distribution of dopamine before and after MRgFUS thalamotomy to gain a deeper understanding of the overall changes induced by this therapy. Additionally, the study suggests exploring sex-specific differences and considering variations in morphology between genders to reduce bias.
Michell, AW et al. [21]	This study used mass spectrometry proteomics to identify a panel of blood biomarkers for early Parkinson’s Disease. The researchers applied a machine learning model to identify PD patients.	The study concludes that clinicians must detect Parkinson’s Disease at early stages. It also highlights the potential of machine learning models to identify the disease up to 7 before motor symptoms arise.	The study advocates for a multivariate approach using state-of-the-art machine learning models and proteomics to validate and potentially apply these findings in future clinical settings.
Hassin-Baer, S et al. [34]	The aim of this study is to explore the potential of biomarkers to differentiate between early-stage Parkinson’s disease and healthy brain function using electroencephalography, event-related potentials, and Brain Network Analytics, with the help of machine learning for data analysis.	The study found that Brain Network Analytics is an effective tool for distinguishing patients with Parkinson’s Disease. The use of machine learning to incorporate event-related potentials was also highlighted.	The article recommends further research with larger and more diverse groups of participants to reduce bias. Additionally, it suggests specific studies focusing on the premotor prodromal phase of Parkinson’s disease in patients.
Maass, F et al. [45]	The manuscript aims to validate the use of a model that can classify Parkinson’s disease patients and age-matched controls based on the levels of specific bio-elements in cerebrospinal fluid. Mass spectrometry and a Support Vector Machine model were used to differentiate between PD and control groups.	The study found that the Support Vector Machine model could successfully distinguish Parkinson’s Disease from control patients within a local cohort. However, its performance was lacking when applied to external cohorts, which attributed to center-specific biases. Nevertheless, the study suggests that bioelemental patterns in CSF could serve as potential biomarkers for Parkinson’s Disease.	The study recommends further research that adheres to more rigorous protocols for pre-clinical and clinical analysis standards, in order to reduce variability and enhance the reliability of bioelemental biomarkers. Additionally, it suggests using mimics in future research to strengthen the model predictions.
Yu, E. et al. [63]	The aim of this study is to identify potential genes associated with Parkinson’s disease through Genome-wide association studies loci. Firstly, all the genes and Single nucleotide polymorphisms are defined. Then, machine learning is used to select genes from different loci.	The study utilized Parkinson’s Disease relevant transcriptomics, epigenomics, and other genetic data sets to develop a boosting model. This model nominated causal genes from Parkinson’s Disease Genome-wide association studies loci, identifying novel genes potentially involved, such as those in the inositol phosphate biosynthetic pathway.	The study recommends further research that addresses the limitations of this study’s development. Specifically, it suggests including a more diverse population, as the study was conducted only in Europeans. It also suggests a broader analysis that includes chromosome X and a wider gene set, not limited to the established Parkinson’s Disease genes.
Costantini G et al. [88]	This article delves into the use of machine learning (ML) and deep learning (DL) models for evaluating vocal characteristics in individuals with Parkinson’s Disease. The study compares both models to determine which approach is the most effective.	The study concluded that both models achieved similar results in classifying Parkinson’s Disease patients based on vocal analysis. K-nearest neighbors slightly outperformed the other models.	This study supports the use of AI as a non-invasive, cost-effective tool for early detection and tracking of Parkinson’s Disease. It emphasizes the importance of collecting high-quality voice data and suggests further research into models that integrate complex neural network architectures.

**Table 2:** Comparative Overview of AI Architectures in Parkinson’s Disease Diagnosis and Severity Assessment.

AI Architecture	Input Modality	Diagnostic Output	Performance Metrics	Metrics Reference
CNN with Attention Mechanism	Nocturnal Breathing Signals	PD Detection and Severity Estimation	AUC: 0.90 (held-out), 0.85 (external)	Yang et al., 2022 [12]
SVM Classifier	18F-FDG PET Radiomic Features	PD vs. Healthy Classification	Accuracy: 90.97%, 88.08%	Wu et al., 2019 [114]
Multi-scale CNN (MCNN)	EEG Signals (PSD & PLV Features)	PD Classification	Accuracy: >99%	Qiu et al., 2022 [115]
AlexNet CNN with TFR	EEG Signals	PD Diagnosis	High Accuracy (Specific metrics not provided)	Li et al., 2024 [116]

## Data Availability

No new data were created or analyzed in this study.

## Abbreviations

The following abbreviations are used in this manuscript:

AI	Artificial Intelligence
ML	Machine Learning
PD	Parkinson’s Disease
DBS	Deep Brain Stimulation
FUS/MRgFUS	(Magnetic Resonance-guided) Focused Ultrasound
SVM	Support Vector Machine
CNN	Convolutional Neural Network
EEG	Electroencephalography
ERP	Event-Related Potential
CSF	Cerebrospinal Fluid
MRI	Magnetic Resonance Imaging
PET/18F-FDG PET	Positron Emission Tomography/18F-fluorodeoxyglucose PET
GWAS	Genome-Wide Association Studies
BDNF	Brain-Derived Neurotrophic Factor
UPDRS/MDS-UPDRS	Unified Parkinson’s Disease Rating Scale/Movement Disorder Society-UPDRS
GBD	Global Burden of Disease
DALY	Disability-Adjusted Life Years
WHO	World Health Organization
GPi	Globus Pallidus Internus
STN	Subthalamic Nucleus
VIM	Ventral Intermediate Nucleus (of the Thalamus)
FDA	U.S. Food and Drug Administration
GDPR	General Data Protection Regulation
HIPAA	Health Insurance Portability and Accountability Act
LRRK2	Leucine-Rich Repeat Kinase 2
PLV	Phase-Locked Value
PSD	Power Spectral Density
AUC	Area Under the Curve (ROC)
ROC	Receiver Operating Characteristic
ICA	Independent Component Analysis
PCA	Principal Component Analysis
NB	Naive Bayes
KNN	K-Nearest Neighbors
GLRLM	Gray-Level Run Length Matrix
GLCM	Gray-Level Co-Occurrence Matrix
WST	Wavelet Scattering Transform
TFR	Time-Frequency Representation
EMR	Electronic Medical Record
ANCOVA	Analysis of Covariance
TMEM175	Transmembrane Protein 175 (Gene)
RAB32	Member RAS Oncogene Family 32 (Gene)
